# Comparison of generalist predators in winter-flooded and conventionally managed rice paddies and identification of their limiting factors

**DOI:** 10.1186/2193-1801-3-418

**Published:** 2014-08-09

**Authors:** Mayura B Takada, Shun Takagi, Shigeki Iwabuchi, Takuya Mineta, Izumi Washitani

**Affiliations:** Institute for Sustainable Agro-ecosystem Services, University of Tokyo, Midori-cho, Nishi-Tokyo, Tokyo, 188-0002 Japan; Department of Environmental Science, Faculty of Science, Toho University, Miyama 2-2-1, Funabashi, 274-8510 Japan; Rice Paddies Japan, Tajiri, Osaki-city, Miyagi, 989-4302 Japan; National Institute for Rural Engineering, Kannondai, Tsukuba-shi, Ibaraki, 305-8609 Japan; School of Agricultural and Life Sciences, University of Tokyo, Yayoi, Bunkyo-ku, Tokyo, 113-8657 Japan

**Keywords:** Biodiversity-friendly farming, Natural enemy, Restoration, Spiders, Waterfowl, Wetland

## Abstract

Winter-flooding of rice paddies without the application of agricultural chemicals is attracting attention as a new agricultural method for enhancing the habitat conditions of wintering waterfowl in rice paddy ecosystems throughout Japan and east Asia. Conditions in these paddies are expected to result in restoration of not only the winter habitats of waterfowl but also those of other taxonomic groups during the rice growing season. In this study, we tested whether the diversity of summer spiders––ubiquitous predators in rice paddies––was higher in the winter-flooded paddies than in the conventional ones by conducting field measurements in 31 winter-flooded and 7 conventional paddies. Limiting factors of spiders in the winter-flooded paddies were then examined. Results revealed that both the density and species richness of spiders were significantly higher in the winter-flooded paddies than in the conventional ones both before and after the insecticide application against pecky rice bug *Stenotus rubrovittatus* (Matsumura)(Hemiptera: Miridae) to conventional paddies. In addition, spider density and species richness in the winter-flooded paddies correlated with the availability of two prey groups––chironomids and other nematocera. These findings suggest that in the winter-flooded paddies the diversity of generalist predators is higher than in the conventional ones during the rice-growing season and that the combination of management at both the landscape and field level is likely more effective for increasing spider abundance in winter-flooded paddies.

## Introduction

Biological communities in agricultural crop fields that have received low or no chemical application are more diverse than those of conventionally farmed fields (e.g. Bengtsson et al. 2005). In particular, rice paddies maintained by biodiversity-friendly farming techniques are expected to function as important substitute habitats for organisms in natural wetlands, which have declined due to the development of urban and agricultural fields (Fasola and Ruiz 1997; Washitani 2007).

The farming practices of biodiversity-friendly paddy fields include the winter flooding of paddies to enhance the habitat conditions for wintering waterfowl (Elphic and Oring 1998; Tourenq et al. 2003; Kurechi 2007). In the paddies at the Ramsar wetland site that surrounds the natural Kabukuri-numa Wetlands, which is one of the most important wintering sites for geese in Japan, the winter-flooding regime combined with no agricultural chemical applications has become a growing trend. This farming method is attracting attention as a new biodiversity-friendly approach throughout Japan and East Asia (Kurechi 2007; Washitani 2007). In fact, to restore paddy biodiversity including wintering waterfowl, no agricultural chemicals have been applied to the winter-flooded (WF) paddies in this region since around 1998. Moreover, WF paddies are also expected to enhance ecosystem services for rice production, including the biological control of insect pests by generalist predators.

In this study, the diversity of spiders––ubiquitous predators in rice paddies––was compared between WF and conventional paddies through field measurements conducted in 31 WF paddies and 7 conventional ones. In addition, factors limiting the abundance of spider populations in WF paddies were also examined. Crop yield in biodiversity-friendly fields can be low compared with that in paddies that receive conventional chemical applications (de Ponti et al. 2012). Thus, effective pest management techniques other than the use of insecticides are needed to sustain growth in biodiversity-friendly farming (Zehnder et al. 2007). Identifying the limiting factors of generalist predators in the WF paddies can help with the construction of effective management plans for insect pest control, because our previous studies showed that the spiders predate insect pests (Kobayashi et al. 2011; Takada et al. 2013), and suppress the density and the consequent damage in organic paddy fields (Takada et al. 2012). Spider population growth is known to be constrained by habitat complexity (Langellotto and Denno 2004; Takada and Miyashita 2004, 2014) and/or prey availability (Wise 1979; Miyashita 1992). In this study, we focused on the vegetation structure of rice and abundant weeds, as well as the biomass of abundant arthropod groups, such as chironomidae and other nematocera, brachycera, and hoppers including Cicadellidae and Delphacidae, and the pecky rice bug *Stenotus rubrovittatus*, as potential limiting factors of the spiders.

## Methods

### Study site

A field survey was conducted in the area surrounding Kabukuri-numa at Osaki City, Miyagi Prefecture, Northern Japan (38°37′N, 141°07′E), where many WF paddies (total area: about 0.2 km^2^) have been cultivated (Kurechi 2007) since around 1998. The mean annual precipitation from 2002 to 2007 was approximately 1166 mm, and the mean temperature in August during the same period was 23.6°C (Japan Meteorological Agency 2012). In this region, insecticides for *Stenotus rubrovittatus* (Hemiptera: Miridae), the most abundant and influential rice pest (Yoshioka et al. 2011; Takada et al. 2012; Yoshioka et al. in press), are applied to most of the conventional paddy fields after heading of rice plants in early August, when spider diversity in paddies is at the highest level (Oyama et al. 2005). Field measurements were conducted twice, just before and after application of the insecticides to conventional fields, with the expectation that spider diversity decreases in conventional paddies, while remaining unchanged in WF ones after the application of insecticide to conventional fields.

In this area, clusters of WF paddies were distributed among conventionally managed paddies. The WF fields have been maintained by using similar management practices that include the application of natural fertilizers, such as fertilizer made from fish parts, and exclude chemical fertilizers, herbicides, and insecticides. Before seedling transplanting in the middle of May, the natural fertilizers are applied to WF fields. Some of the fields that did not receive herbicide application were dominated by weeds, such as *Monochoria vaginalis* var. *plantaginea*. So farmers do the mechanical weeding once or twice in June*.* WF fields are flooded all year. Even in winter (not rice-growing season) WF fields are flooded for providing suitable habitats for waterfowl (Kurechi 2007). A typical farming schedule in the conventional paddies is as follows. Fungicides (Tetrachloroisophthalonitrile or hydroxyisoxazole and Metalaxyl) are applied to young rice plants in seedling boxes, and herbicides (indanofan, bensulfuronmethyl, and clomeprop (5 kg ha^-1^) or Pyriminobac-methyl, bromobutide, bensulfuronmethyl, and pentoxazone (10 kg ha^-1^)) are applied directly to the field after seedling transplanting in early May. Insecticides (etophenprox (2-3 l ha^-1^)) for rice leaf beetle *Oulema oryzae* Kuwayama are applied to the fields and then water is drained from the field for about ten days to stabilize rice yield and increase soil hardness in early June. After heading of rice plants in early August, insecticides for pecky rice bugs (dinotefuran (30 kg ha^-1^)) are applied once or twice to the field. The insecticides are applied to many of the conventional rice fields by radio-controlled helicopters. Most conventional paddies are seasonally dried-up after harvesting.

### Field survey

A total of 31 WF and 7 conventional paddies were selected in an area of approximately 20 km^2^. The mean (SD) area of the study fields was 2100.8 ± 438.2 m^2^ and 1780.3 ± 523.7 m^2^ for the WF and conventional fields, respectively. Rice seedlings were transplanted in May 2007 at a mean (SD) density of 21.20 ± 2.0 hills/m^2^ and 23.24 ± 2.0 hills/m^2^ in the WF and conventional fields, respectively. In August 2007, net samplings (20 sweeps with a 36-cm-diameter insect net) for spiders and their potential prey were performed at the center of each field just before and after using insecticides for pecky rice bugs in conventional fields (insecticide application: 6, 18–19 August, first survey: 1–4 August; Second survey: 15–19 August). The sweeping sampling is an excellent method to collect invertebrates inhabiting the uppermost vegetation layer in paddies easily. Invertebrate body mass was calculated as mass (mg) = 0.0305 × (body length (mm))^2.62^ (Rogers et al. 1976) to measure the abundance of potential prey for spiders. As indices of habitat complexity, we measured rice height of six randomly chosen hills and counted the number of individuals of the most abundant weed (*M. vaginalis*) within a 1 × 3 m^2^ quadrant at the center of each field (e.g., Rypstra et al. 1999, Miyashita and Takada 2007).

### Statistical analysis

To evaluate the effects of the farming practices (WF vs. conventional) and the insecticides applied to conventional fields (before vs. after) on the numbers of individual spiders and spider species in paddies of both types, we applied generalized linear mixed effect models with Poisson error distribution because the dependent variables are count data. Fixed factors in the models were the main effects of the farming practice and the insecticide applications and their interaction, and the random factor was the effect of the paddy. The significance of each fixed effect was checked by using analysis of deviance and by checking for the reduction in residual deviance by reducing each effect by means of the Chi-squared test.

The factors limiting the density and diversity of spiders in the WF paddies were analyzed by using generalized linear mixed effect models with Poisson error distribution because the dependent variables are count data. We examined the limiting factors of spider density and species richness using only the data of the first survey to avoid redundant analyses, because spider density in the WF paddies tended to be higher in the first field survey than in the second (see Results) and the results obtained from preliminary analyses using the data of the second survey were similar to those of the first survey. This model included the number of individual spiders or spider species richness as the dependent variable, and two indices of habitat complexity (mean rice height and *M. vaginalis* density) and the biomass of five prey groups as fixed factors (Table 1), with the paddy field as a random effect. All tolerance values between the fixed factors were greater than the critical value of 0.1 (Table 1), indicating no significant collinearity between them (Quinn and Keough 2002). Model selection for the generalized linear mixed effect models was performed using Akaike’s Information Criterion (AIC). AIC was calculated for each possible combination of fixed factors, and the models with small AIC were chosen as optimal models. All statistical analysis was carried out using *R* for Windows 2.13.1 (*R* Development Core Team 2011).

**Table 1:** **Independent variables used in the generalized linear mixed effect models for identifying limiting factors of spider density and species richness in winter-flooded (WF) paddies**

Independent variables	Mean	Range	Tolerance values
Habitat complexity			
Mean rice height (cm)	67.34	59.67-77.83	0.903
*Monochoria vaginalis* (no.individuals/3 m^2^)	67.42	0-393	0.794
Prey availability (mg/20 sweeps with an insect net)			
Chironomid	130.68	14.23-975.86	0.837
Other nematocera	32.07	6.58-100.54	0.913
*S. rubrovittatus*	7.08	0-65.37	0.746
Brachycera	5.23	0.38-29.90	0.831
Hopper	3.98	0.79-10.17	0.823

## Results

A total of 234 spiders in 15 species or families were observed. Dominant spider species were *Tetragnatha caudicula*, *T. praedonia,* and *T. extensa* for web-building spiders and *Pachygnatha quadrimaculata*, *P. clercki*, and Lycosidae spp. for cursorial spiders (Table 2). Juveniles were identified only to family.

**Table 2:** **Mean density of each spider species or family per field for WF and conventional paddies**

Species/family	Before insecticide application		After insecticide application	
	WF	Conventional	WF	Conventional
*Tetragnatha caudicula*	**2.774**	**0.143**	**0.406**	**0**
*T. praedonia*	**0.774**	**0.143**	**0.469**	**0**
*T. extensa*	**0.194**	**0.143**	**0.0406**	**0**
*T. maxillosa*	**0.484**	**0**	**0.094**	**0**
*T. squamata*	**0.097**	**0**	**0.031**	**0**
*Pachygnatha quadrimaculata*	**0.516**	**0**	**0.406**	**0**
*P. clercki*	**0.161**	**0**	**0.344**	**0**
**Lycosidae**	**0.065**	**0**	**0.250**	**0**
*Neoscona adianta*	**0.065**	**0**	**0**	**0**
*Argiope bruennichii*	**0.032**	**0.286**	**0**	**0**
*Larinioides cornutus*	**0**	**0**	**0.063**	**0**
**Linyphiidae**	**0.032**	**0**	**0.281**	**0**
**Thomiside**	**0.032**	**0.143**	**0.031**	**0**
**Theridiidae**	**0**	**0**	**0.031**	**0**
**Unknown spiders**		**0**	**1.781**	**0.143**

**Figure 1:**
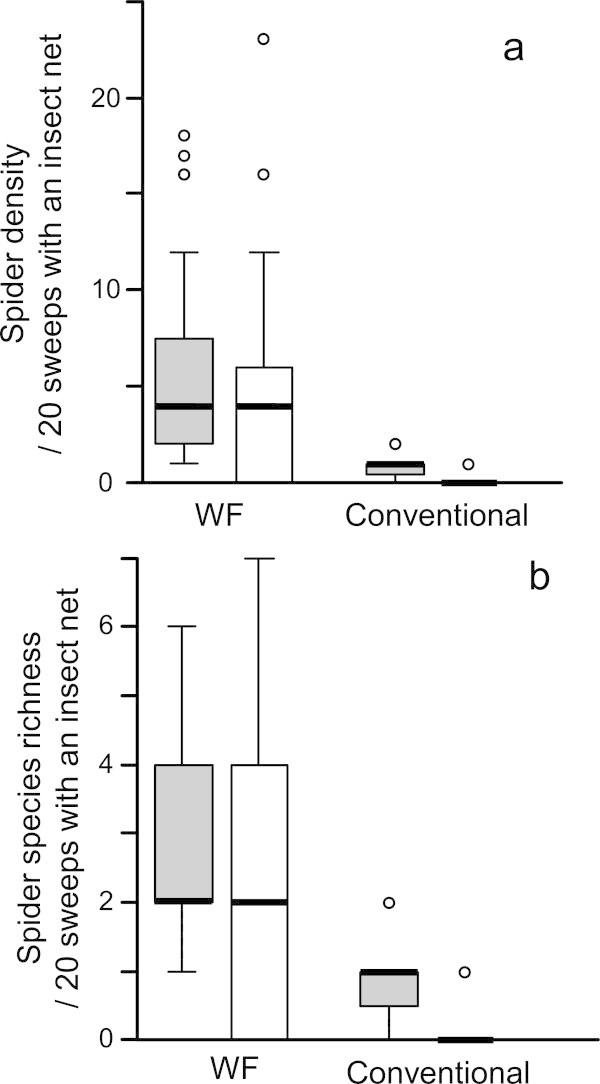
**Spider diversity in WF and conventional paddy fields.** Boxplots for the spider density **(a)** and species richness **(b)** in the WF and conventional paddy fields before (filled bars) and after (open bars) applying insecticide to conventional paddies in early August.

The main effect of farming practice (WF vs. conventional) on spider density (χ^2^_1_ = 23.369, *P* < 0.001) and species richness (χ^2^_1_ = 17.817, *P* < 0.001) was highly significant, while the interaction effect of farming practice and insecticide application was significant on neither spider density nor species richness (density: χ^2^_1_ = 2.792, *P* = 0.095; species richness: χ^2^_1_ = 3.204, *P* = 0.073). This result indicates that spider density and species richness were higher in WF paddies than in conventional ones both before and after insecticide applications to conventional paddies (Figure 1). Since the main effect of insecticide application (before vs. after) was significant only for spider density (density: χ^2^_1_ = 5.920, *P* = 0.015; species richness: χ^2^_1_ = 1.417, *P* = 0.234), insecticide application to conventional paddies was suggested to reduce spider density in both the WF and conventional paddies (Figure 1). Moreover, the variations in spider density and species richness in the WF paddies seemed to increase after applying insecticide: the interquartile range––the difference between upper (75% quartile) and lower (25% quartile) hinges––of spider density was 5.5 before insecticide application, but 6 after (Figure 1a), and the interquartile range of spider species richness was 2 before insecticide application, but 4 after insecticide application (Figure 1b).

**Figure 2:**
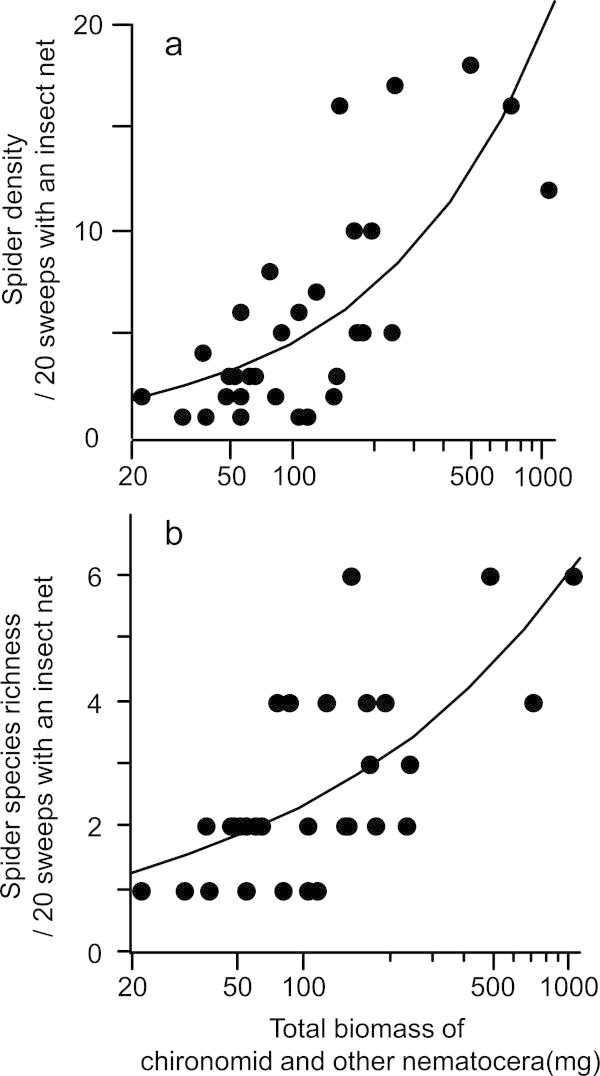
**Spider diversity and their potential prey in WF paddy fields.** Relationships between total biomass of important prey (chironomids and other nematocera) and spider density **(a)** and species richness **(b)** in WF paddies before applying insecticide to conventional fields. Poisson regression lines are also shown.

The best model for explaining the spider density and species richness in WF paddies included the biomass of chironomids and other nematocera (Table 3) but did not include any factors of habitat complexity, such as mean rice height or *M. vaginalis* density. These two groups of prey increased both spider density and species richness (Figure 2).

**Table 3:** **Estimated coefficients(SE) and information theoretic statistics of the top five and null (intercept only) models explaining spider density and species richness in WF paddies**

Rank	Intercept	Rice height	***M. vaginalis***	Chironomid	Other nematocera	***S. rubrovittatus***	Brachycera	Hopper	AIC	Δ AIC	***w***_***i***_
Spider density											
1	-0.941 (0.457)	-	-	0.473 (0.092)	0.014 (0.004)	-	-	-	52.10	0	0.119
2	-0.717 (0.471)	-	-	0.460 (0.090)	0.013 (0.004)	-	-0.028 (0.022)	-	52.45	0.34	0.100
3	0.844 (1.736)	-0.027 (0.26)	-	0.487 (0.93)	0.013 (0.004)	-	-	-	52.97	0.87	0.077
4	-0.755 (0.543)	-	-0.031 (0.53)	0.445 (0.0102)	0.014 (0.004)	-	-	-	53.75	1.65	0.052
5			-	0.473 (0.091)	0.013 (0.004)	-	-0.025 (0.023)	-	53.80	1.70	0.051
Null	1.490 (0.164)		-	-	-	-	-	-	73.44	21.33	0
Spider species richness											
1	-0.535 (0.481)	-	-	0.278 (0.098)	0.008 (0.005)	-	-	-	21.13	0	0.074
2	-0.286 (0.517)	-	-	0.262 (0.097)	0.009 (0.005)	-	-	-0.053 (0.044)	21.63	0.50	0.057
3	-0325 (0.451)	-	-	0.294 (0.096)	-	-	-	-	21.87	0.74	0.051
4	-0372 (0.515)	-	-	0.268 (0.099)	0.007 (0.005)	-	-0.020 (0.25)	-	22.40	1.27	0.039
5	-0.138 (0.481)	-	-	0.278 (0.097)	-	-	-0.025 (0.025)	-	22.73	1.60	0.033
Null	0.960 (0.111)	-	-	-	-	-	-	-	28.68	7.55	0.002

Ranking of the sub models is based on Akaike’s information criterion.

## Discussion

We revealed that spider density and species richness were higher in the WF paddies than in the conventional ones both before and after insecticide application to conventional paddies for pecky rice bugs in early August. Moreover, our results suggest that the insecticide application decreased spider density in not only conventional paddies but also WF ones. The spider diversity in the WF paddies was significantly affected by the availability of two prey groups, chironomids and other nematocera.

The significant main effect of farming practice (WF vs. conventional) on spider density and species richness appears to indicate that these values were already lower in conventional fields than in WF ones before the insecticide application to conventional paddies (Table 2, Figure 1). At least four possible explanations for the farming practice effect can be identified. First, fungicides applied to young rice plants in seedling boxes, herbicides and insecticides early in the rice-growing season may be responsible for the reduced spider diversity in conventional fields during the early-growing season. Such agricultural chemicals are known to affect invertebrates (Oyama and Kidokoro 2003; Wilson et al. 2008; Amano et al. 2011) and were probably responsible for decreasing the number of spider prey in the paddies. The second mechanism for the farming practice effect on the spiders is due to the drainage of conventional paddies in early June. Drainage dries and hardens the soil in conventional paddies (see Study site) and likely decreases the number of aquatic invertebrate, including juveniles of dipterous insects, which are important prey for spiders (Ishijima et al. 2006; Tahir and Butt 2009). Third, organic fertilizer applied to the WF fields before seedling transplanting might increase spider diversity due to increasing the availability of important prey for spiders. Previous studies have reported that detrital subsidy, such as organic fertilizer, enhances spider populations indirectly through increasing detritivores, such as chironomids and collembolas (Murata 1995; Settle et al. 1996; Halaj and Wise 2002). Finally, tubificid worms as well as waterfowl fertilize WF paddy soil with their feces (Kurechi 2007), which may increase spider diversity due to increasing the availability of important prey for spiders. Because WF fields are flooded all year, tubificid worms are known to active even in winter, fertilizing the soil in WF fields. On the other hand, conventional fields are dried-up after harvesting. Clarification of these causalities requires manipulative experiments controlling each component with many replications.

The main effect of insecticide application (before vs. after) on spider density was also significant, suggesting the insecticides applied to conventional fields may decrease spider density in not only conventional but also WF paddies, despite not applying insecticides to WF paddies directly. One possible explanation is as follows. WF and conventional paddy fields are spatially intermingled in the study area, so insecticides for pecky rice bugs sprayed onto the conventional rice fields by radio-controlled helicopters may easily diffuse into adjacent WF paddies and consequently affect spider density. Increased variation in the spider density and species richness in WF paddies after insecticide application (Figure 1) appears to support this possibility, because the intensity of the negative effect of the insecticides on spiders inhabiting the WF paddies may vary depending on the distance between the WF and conventional paddies. If so, increasing the spatial gathering of WF paddies will lead to an increase in the number of spiders in each WF paddy due to avoiding the diffusion of insecticides sprayed by radio-controlled helicopters. Enhancement of spider populations by strategic spatial placement of WF paddies may also suppress damage by pecky rice bugs in WF paddies. Spider species with high abundance in the WF paddies were also shown to predate pecky rice bugs through previous DNA-based gut-content analysis (Kobayashi et al. 2011; Takada et al. 2013). Moreover, *Tetragnatha* spp. spiders were revealed to suppress bug density and the consequent damage in organic paddies fields (Takada et al. 2012).

Spider diversity in WF paddies increased with the increasing abundance of chironomids and other nematocera. This was revealed by statistical analysis of the limiting factors of spider diversity (Table 3). Because we used a comparative approach, the casual relationship between spiders and these insects was no proven in this study. However, previous studies have suggested that these dipterous insects are frequently consumed by the spiders in paddies (Ishijima et al. 2006; Tahir and Butt 2009). Taken together, the dipterous insects most likely limit spider diversity in WF paddy fields. Our study is one of the few that provides evidence of the correlation between the abundance of important prey and increased spider diversity in paddies, with many replications.

Spiders and their potential prey were collected only by one method (i.e. sweeping sampling) in this study. Sweeping sampling is an excellent method to collect invertebrates inhabiting the uppermost vegetation layer in paddies easily. But it is necessary to use other methods (e.g., direct counting), to estimate spiders inhabiting the bottom layer, such as Lycosidae.

Many studies have shown that flooding rice paddies can provide suitable habitats for waterfowl (Elphick and Oring 1998; Tourenq et al. 2003; Kurechi 2007). The present study suggests that in WF paddies the diversity of generalist predators is higher than in conventional paddies during the rice-growing season. Moreover, a combination of management at both the landscape and field level seems to be more effective for increasing spider diversity in WF paddies. Spiders are not only important natural enemies of various insect pests (Kiritani et al. 1970; Oraze et al. 1989; Takada et al. 2012), but also important prey for larger predators, such as frogs (e.g., Hirai and Matsui 2001) and larger spiders (e.g., Ishijima et al. 2006) in paddies. Taken together, the enhancement of spider diversity in WF paddy fields might achieve both biological control and biodiversity conservation in rice paddy ecosystems.
